# A new method to measure higher visual functions in an immersive environment

**DOI:** 10.1186/1475-925X-13-104

**Published:** 2014-07-28

**Authors:** Giuseppe A Zito, René Müri, Urs P Mosimann, Thomas Nyffeler, Tobias Nef

**Affiliations:** 1 Gerontechnology and Rehabilitation Group, University of Bern, Bern, Switzerland; 2 Division of Cognitive and Restorative Neurology, Department of Neurology, University Hospital Inselspital, University of Bern, Bern, Switzerland; 3 University Hospital of Old Age Psychiatry and Psychotherapy, University of Bern, Bern, Switzerland; 4 Center of Neurology and Neurorehabilitation, Luzerner Kantonsspital, Luzern, Switzerland; 5 ARTORG Center for Biomedical Engineering Research, University of Bern, Bern, Switzerland

**Keywords:** Computer-based test, Distortion algorithm, Hemispherical projection, Unilateral brain lesion, Visual perception

## Abstract

**Background:**

Higher visual functions can be defined as cognitive processes responsible for object recognition, color and shape perception, and motion detection. People with impaired higher visual functions after unilateral brain lesion are often tested with paper pencil tests, but such tests do not assess the degree of interaction between the healthy brain hemisphere and the impaired one. Hence, visual functions are not tested separately in the contralesional and ipsilesional visual hemifields.

**Methods:**

A new measurement setup, that involves real-time comparisons of shape and size of objects, orientation of lines, speed and direction of moving patterns, in the right or left visual hemifield, has been developed. The setup was implemented in an immersive environment like a hemisphere to take into account the effects of peripheral and central vision, and eventual visual field losses. Due to the non-flat screen of the hemisphere, a distortion algorithm was needed to adapt the projected images to the surface. Several approaches were studied and, based on a comparison between projected images and original ones, the best one was used for the implementation of the test. Fifty-seven healthy volunteers were then tested in a pilot study. A Satisfaction Questionnaire was used to assess the usability of the new measurement setup.

**Results:**

The results of the distortion algorithm showed a structural similarity between the warped images and the original ones higher than 97%. The results of the pilot study showed an accuracy in comparing images in the two visual hemifields of 0.18 visual degrees and 0.19 visual degrees for size and shape discrimination, respectively, 2.56° for line orientation, 0.33 visual degrees/s for speed perception and 7.41° for recognition of motion direction. The outcome of the Satisfaction Questionnaire showed a high acceptance of the battery by the participants.

**Conclusions:**

A new method to measure higher visual functions in an immersive environment was presented. The study focused on the usability of the developed battery rather than the performance at the visual tasks. A battery of five subtasks to study the perception of size, shape, orientation, speed and motion direction was developed. The test setup is now ready to be tested in neurological patients.

## Background

Visual perception is a complex process. In the brain, the primary visual cortex (V1) receives input arriving in an orderly manner from the retina. The properties of objects, their shape, color, motion and location in the world, are then represented beyond V1 in specialized extrastriate areas [1,2]. It is here that the brain assesses the crucial defining qualities of objects and collects, processes and collates visual information to form a coherent whole. This high-level visual perception embraces the complex network of higher visual functions (HVF).

It is known that an impairment of HVF after unilateral brain injury may influence the perception of the visual world [3], for example the capacity to recognize visually presented objects (visual agnosia) [4] or the capacity to perceive visual motion (akinetopsia) [5]. Impaired HVF are usually tested with paper pencil tests. Examples of such tests are the “Visual Objects and Space Perception battery” (VOSP) [6], where the test persons are asked to name visually presented objects, distinguish them from nonsense pictures and recognize the spatial location of figures, or the “Birmingham Object Recognition Battery” (BORB) [7], where images of animals together with meaningless figures are presented to the participants and they have to identify the meaningful ones. Another common test used for spatial perception is the “Benton judgment for line orientation” [8], in which the subjects compare straight lines oriented in different directions with a pull of reference lines and they have to find the line in the pull that matches the orientation of the line under test. Motion blindness is often tested with Random Dot Cinematograms [9], patterns of dots moving in a direction that has to be recognized by the subjects. In all these tests, the results are usually the number of items correctly addressed at the different tasks and the diagnosis is given based on this number and on a comparison with normative data.

However, there are no computer-based tests that return quantitative data as indicators for the magnitude of unilateral impairment. Furthermore, standard neurological tests do not discriminate objects perceived in the right visual hemifield and in the left one and, thus, the degree of dynamic interaction between the two brain hemispheres. Therefore a new measurement paradigm (HVF test battery) involving real-time comparison of images in the two visual hemifields was developed and piloted in healthy volunteers.

The HVF test battery was implemented in an immersive environment such as a hemispherical screen, whose peculiar characteristic is a field of view of ± 90° in both horizontal and vertical axes. Since HVF disorders are often related to visual field defects [10], the hemispherical screen presents the advantage of testing HVF in the region of the visual field that is still intact. Furthermore it allows for consideration of the effects of central vision and peripheral vision while accomplishing the tasks.

In order to implement the HVF test battery, interactive images need to be projected onto a non-flat surface. Several methods were analysed [11], then a mathematical model and a fully measured approach were implemented to pre-distort images to look undistorted once they are projected onto the hemispherical screen. The two methods were compared and the best solution was selected for the final implementation.

The pilot study on the HVF test battery was at last conducted. The aims were to assess the performance level at the proposed tasks in case of intact HVF, as well as to collect feedback from the participants regarding the usability of the battery itself.

## Methods

### Hardware

**Figure 1:**
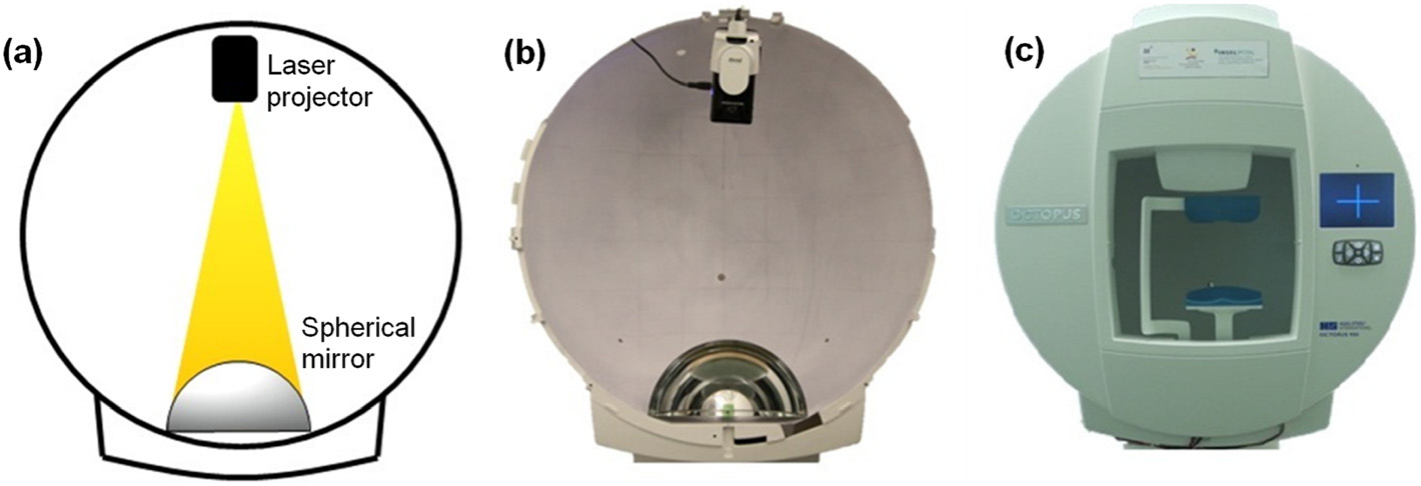
**Hardware setup for the implementation.** Mechanical setup for the immersive environment. **a)** Scheme of the projection system with the projector in the upper part and the spherical mirror in the lower part. **b)** Front view of the perimeter used for the implementation, without the main panel. **c)** Front view of the perimeter, with the main panel.

In order to achieve an immersive environment of ±90° in both horizontal and vertical directions, a hemispherical projection screen (cupola) with a diameter of 60 cm was selected. The cupola was placed on a height-adaptable table (73 – 93 cm) and equipped with a motorized chin- and forehead-rest (±2 cm in vertical and horizontal directions) to let the test person comfortably sit in front of it, with the head positioned in the center of the hemisphere. The correct position of the head at the center of the hemisphere was achieved by means of an infrared camera integrated into the cupola, pointing at the right eye, and whose deviation from the center of the cupola was assumed equal to half the average interpupillary distance (dp = 6.35 cm [12]). The same infrared camera, together with an infrared-pass filter and two infrared LEDs to enlighten the eye, was used to track the gaze position of the test person [13]. For convenience, the setup was implemented by modifying a commercially available perimeter (Octopus 900, Haag-Streit AG, Köniz, Switzerland). This was advantageous because the perimeter is already equipped with a motorized chin- and forehead-rest, a hemisphere of the desired diameter, and an integrated infrared camera with two infrared LEDs (Figure 1c).The images needed to perform the tasks were projected onto the cupola’s own surface by using a small laser projector (MicroVision SHOWWX + Laser Pico Projector) and a spherical mirror with a diameter of 23 cm. The laser projector was placed in the upper part of the cupola, while the spherical mirror in the lower part, as described in Figure 1a.

The laser projector represents an advantage because it projects images in focus in all their pixels onto the cupola, even if the distance projector-screen varies point by point. The spherical mirror also plays a crucial role because it allows for coverage of the entire projection surface and the whole structure to be globally compact.

### Software

In order to achieve projected images which look undistorted onto the cupola, a warp algorithm was needed [12]. Two approaches were implemented and compared.

A mathematical model was previously investigated to warp images [13]: given the geometry of the system, the model computes, per each point on the cupola, where the corresponding point is reflected onto the spherical mirror and, therefore, where it is located on the projector. Then it distorts the images accordingly to the new computed locations of the points.

**Figure 2:**
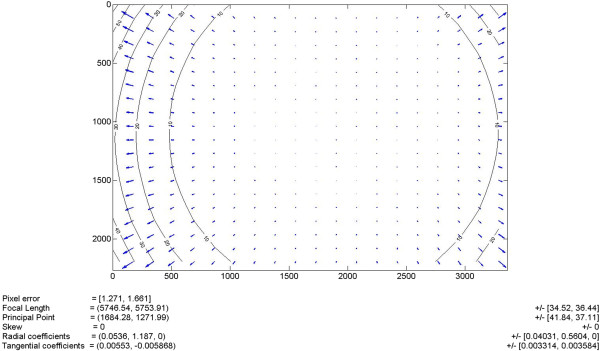
**Fisheye effect.** Fisheye lens distortion of the camera: the blue arrows represent the direction and amplitude of the distortion, computed in different points on the lens of the camera. The calibration parameters are calculated according to the fisheye distortion model described in [14].

**Figure 3:**
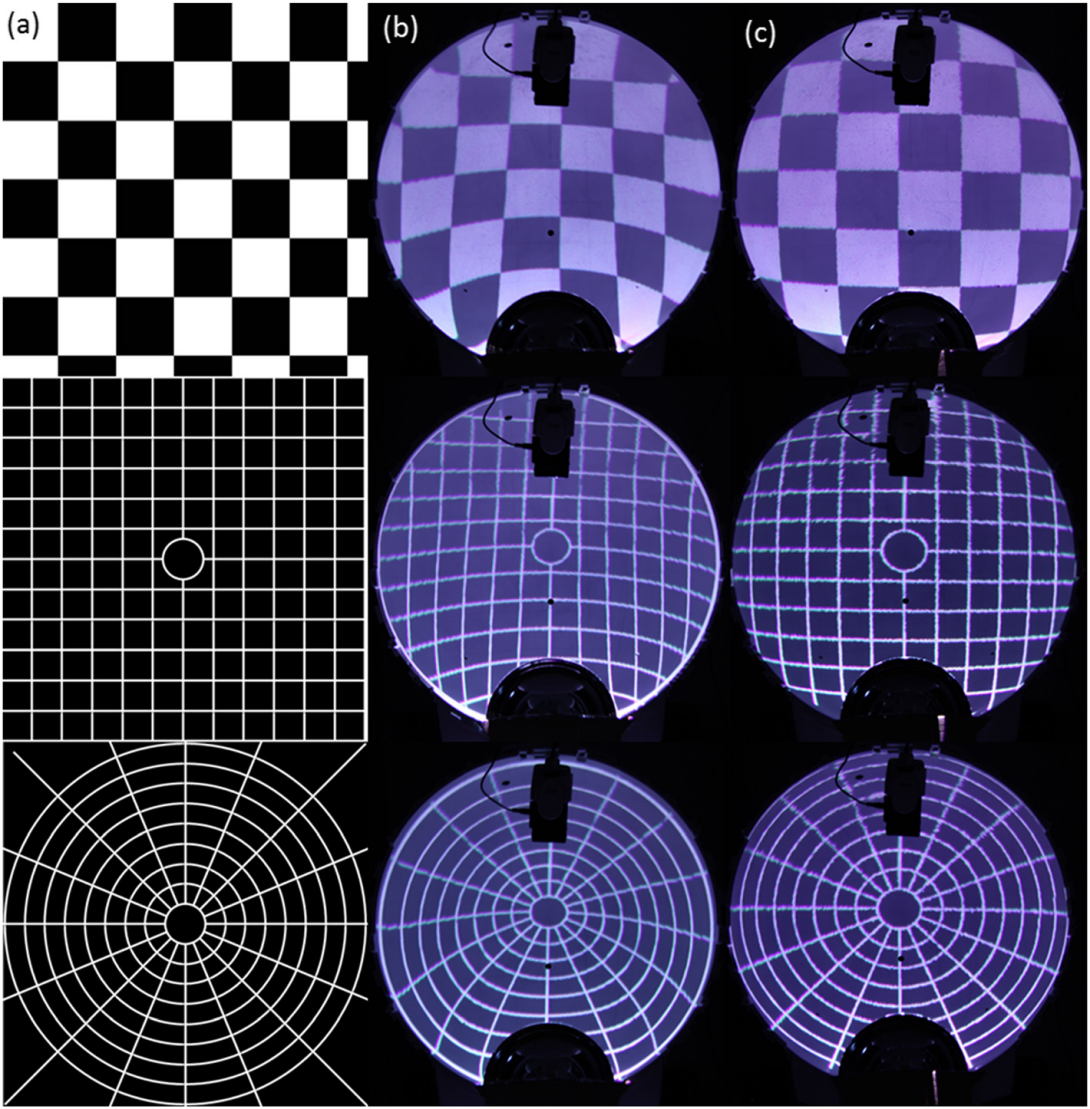
**Reference images, original and projected.** Reference images used for the comparison of the two projection approaches. **a)** Original images. **b)** Images distorted with the mathematical model and projected onto the cupola. **c)** Images distorted with the fully measured approach and projected onto the cupola.

A fully measured approach, where each pixel of the projector is individually sent to the spherical mirror and its location onto the cupola is registered by an external camera (Canon EOS 7D), was also evaluated. In this method the external camera was placed in front of the cupola, directed toward its center and at 2 m distance from it. Its precise position was measured with a grid drawn on the floor of the examination room and its orientation toward the hemisphere was achieved by means of a laser beamer placed in the center of the cupola and pointed at the objective of the camera. The fisheye effect of the lens of the camera was quantified (Figure 2) and corrected, according to [14], with the Camera Calibration Toolbox for Matlab (The MathWorks, Inc.). The fully measured approach applies an inverse transformation simply based on the location of the pixels in two coordinates system, the one of camera (CS_C_), a Cartesian system with the origin at the top-left corner that represents the space where we perceive the images as undistorted, and the one of the projector (CS_P_), also a Cartesian system with the origin at the top-left corner where the images must be pre-distorted. Each pixel in CS_P_ is matched with the corresponding point in CS_C_ and a transformation matrix is computed. Then the match between the two coordinate systems is applied: each pixel in CS_C_ contains five information values, the x and y coordinates, and the RGB values of the image in correspondence of the pixel itself. The transformation matrix moves the x and y values from CS_C_ to CS_P_, while the RGB values remain constant. Once all the pixels are processed, in CS_P_ there is the pre-distorted image.In order to compare the two methods, reference images (in jpeg format, with an original resolution of 720 × 720 pixels) were pre-distorted and projected onto the cupola (Figure 3a).

The projected images were captured by the external camera and the two outputs were correlated, respectively, to the original image. In particular the structure similarity index (SSIM) [15,16] was used to measure the fidelity of the distortion. Since the distortion only affects the structure of the objects in the scene and not the brightness, the SSIM resulted as a good choice to explore the structural information in the images separating it from the influence of the luminosity of the light source (projector).

### Participants and ethical approval

57 healthy volunteers (32 men and 25 women aged 22 to 73) were recruited to participate in this study (age M = 36.1; SD = 12.9). All subjects had a Master Diploma at least, a visual acuity > 0.8, corrected with lenses if needed, and a MoCA score > 26. Moreover they were all used to handling computers.

The usability testing was carried out in accordance with the latest version of the Declaration of Helsinki. Ethical approval was given by the Ethics Committee of the Canton of Bern, Switzerland.

### Test setup

**Figure 4:**
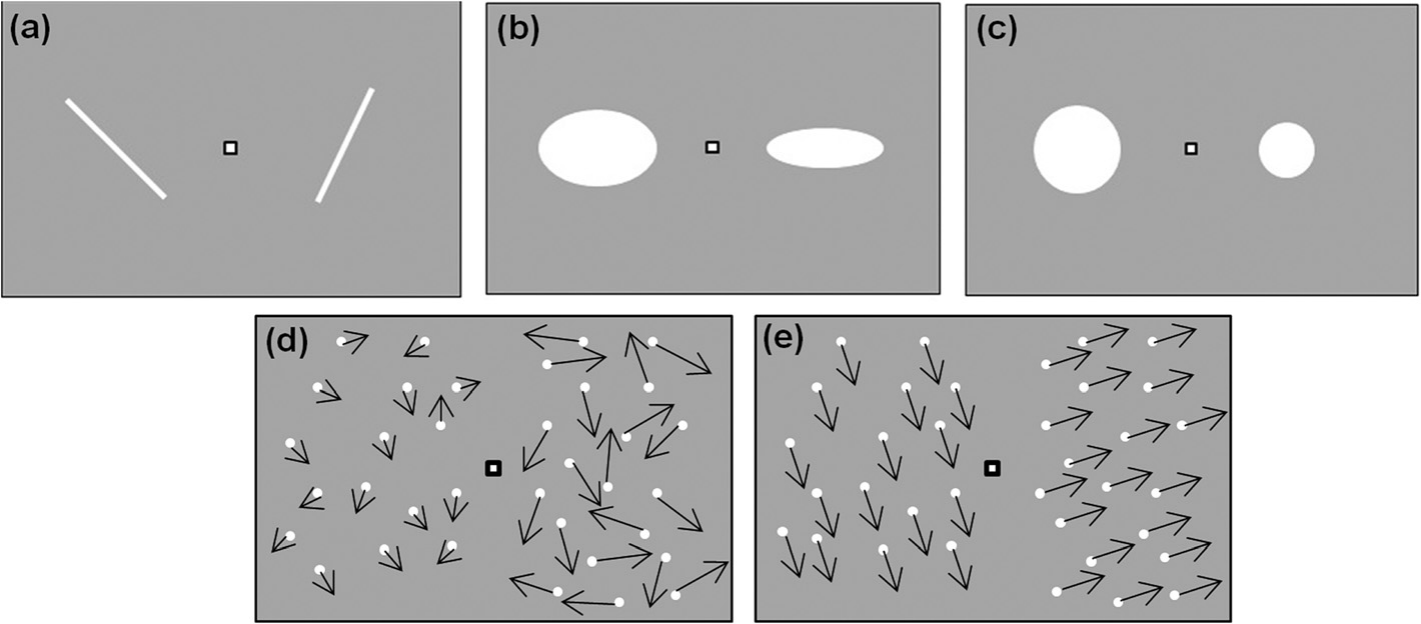
**HVF test battery.** Screenshots from the HVF test battery. **a)** Subtask for line orientation. **b)** Subtask for shape perception. **c)** Subtask for size perception. **d)** Subtask for direction of motion. **e)** Subtask for speed perception.

The HVF test battery consists of five subtasks, where the perception of size, shape, orientation, speed and motion direction is assessed. The tests follow a balance paradigm, in which the test person, sitting in front of the cupola, with the head in the center of the hemisphere and at a distance of 30 cm from the projection surface, has to compare two images that are presented, respectively, on the left and right visual hemifields at 20° eccentricity from the center of the cupola, while fixating at a marker point at 0° eccentricity. The test person holds in his hand an input device with three response buttons, two of them to balance the images and one to confirm the choice. For a perceived continuous balance of the images, the step size of the balancing buttons was selected 10 times smaller than the resolution of the human eye in the fovea, 0.016 visual degrees (°V) [17], in particular, it was 10^−3^ ° for the orientation task, 10^−3^ °V for the shape task, 10^−3^ °V for the size task, 10^−3^ °V/s for the speed task and 10^−3^ ° for the motion direction task. The subject is asked to perform five different subtasks, in a randomized order:1. Orientation Task: two lines, oriented in different directions, are presented; the subject has to rotate, by pressing the balance buttons, the line on the left until it is perceived as parallel as the line on the right (Figure 4a).2. Shape Perception Task: two ellipses, different in geometric eccentricity (ratio between the two axes), are presented; the subject has to change the eccentricity of the ellipse on the left until it is perceived as identical as the one on the right (Figure 4b).3. Size Perception Task: two circles, different in size, are presented; the subject has to change the size of the circle on the left until it is perceived as identical as the one on the right (Figure 4c).4. Speed Perception Task: two patterns of random dots moving with different speed and random directions are presented; the subject has to change the speed of the dots moving on the left until they match the speed of the dots on the right (Figure 4d).5. Task for Perception of Motion Direction: two patterns of random dots moving into different directions are presented; the subject has to change the direction of motion of the entire pattern on the left until it moves in the same direction as the pattern on the right (Figure 4e).

All the five subtasks are performed under central fixation [18], which is controlled using the infrared camera and the infrared LEDs integrated into the perimeter device. If the test person moves his gaze out of an allowed region of ± 1° centered on the fixation marker, the images under balance disappear and they appear again when the marker is fixating again.

The presentation of the images and the recorded answers were processed on a Intel® Core™ i5 (3.10 GHz) with Windows 7 operating system (Microsoft Inc.) and within a Matlab script using the Psychophysics toolbox [19]. The monitor of the computer was a 24’ screen with resolution of 1920x1080 pixels.

### Test procedure

Prior to the examination all participants gave written informed consent. After performing several training sessions to become familiar with the tasks, the subjects started the real measurement (about 20 min), in which five repetitions per subtask were made. The experiments were carried out in a dark, noise-controlled room.

After completing the tests, each participant filled in a Satisfaction Questionnaire (SQ), based on a combination of the ASQ (After Scenario Questionnaire) [20] and the NASA Task Load Index (TLX) [21]. The SQ included ten questions, the first six belong to the NASA TLX, while questions 7–9 belong to the ASQ, question 10 assesses the face validity, the relevance of the test as it appears to the participants [22], with regards to everyday life activities in relation to HVF (Table 1). In each question the response was given on a 7 Likert scale, where 7 corresponded to “very high” and 1 to “very low”.

**Table 1:** Satisfaction questionnaire

**Questions**	**Mean ± SD**
1. How mentally demanding were the tasks?	3.3 ± 1.5
2. How physically demanding were the tasks?	2.6 ± 1.9
3. How hurried or rushed was the pace of the tasks?	2.6 ± 1.3
4. How successful were you in accomplishing what you were asked to do?	4.7 ± 1.5
5. How hard did you have to work to accomplish your level of performance?	4.3 ± 1.7
6. How insecure, discouraged, irritated, stressed and annoyed were you?	1.8 ± 1.2
7. Overall, how satisfied are you with the ease of completing the tasks?	5.5 ± 1.3
8. How satisfied are you with the amount of time it took to complete the tasks?	5.6 ± 1.5
9. How satisfied are you with the support information when completing the tasks?	6.1 ± 1.4
10. How confident are you that the tests measure skills you need in everyday life activities?	5.0 ± 1.6

List of the 10 questions of the Satisfaction Questionnaire, including the responses (mean ± standard deviation) from the participants on a 7 Likert scale, where 7 corresponds to “very high” and 1 to “very low”.

### Data analysis

The outcome measures of the HVF test battery, per each subject, were the mean values, out of the five repetitions, of the absolute differences between the reference images and the images under manipulation at the very time when the confirmation button was pressed. In particular, for the line orientation subtask, it was the angle between the two oriented lines, for the shape perception subtask the difference in eccentricity of the ellipses, for the size perception subtask the difference between the radii of the circles, for the speed perception subtask the difference between the speeds of the patterns, and for the perception of motion direction subtask the angle between the two directions of motion of the patterns.

The outcome of the SQ was the level of agreement of the participants to the questions listed in Table 1.

## Results

### Quality of the distortion

Figure 3b and c show the images distorted with the mathematical model and with the fully measured approach, respectively, in comparison with the original reference images. The computed SSIM per each image is shown in Table 2. For all the images, the fully measured approach returned values for the SSIM index globally higher than the ones from the mathematical model, confirming that the fidelity of the distortion is better with the measured method.

**Table 2:** SSIM index

	**SSIM**	
	**Mathematical model**	**Fully measured approach**
Image 1	94.55%	97.79%
Image 2	98.79%	99.06%
Image 3	98.75%	99.07%

SSIM index calculated for three different images distorted with the mathematical model and the fully measured approach, respectively and compared with the original undistorted images.

### Performance at the HVF test battery

The results of the pilot study, summarized in Table 3, showed that healthy subjects are able to discriminate small differences in size and shape of objects presented in the right visual hemifield and in the left one with an accuracy of 0.18°V and 0.19°V, respectively. Regarding the orientation of visually presented lines, the accuracy was 2.56°, while for motion perception the finest difference was 0.33°V s^−1^ for objects moving at different speeds and 7.41° for patterns moving in different directions.

**Table 3:** Results of the pilot study divided into the five subtasks

**Sub-tests**	**Final difference (mean)**	**Final difference (SD)**
Orientation (N = 57)	2.56°	1.62°
Shape (N = 57)	0.19°V	0.15°V
Size (N = 57)	0.18°V	0.12°V
Speed (N = 57)	0.33°V *s*^−1^	0.25°V *s*^−1^
Direction (N = 57)	7.41°	5.67°

Results of the pilot study divided into the five subtasks. The level of accuracy of the 57 healthy volunteer is expressed in polar angles (°), for the Orientation and Motion Direction subtasks, and in visual angles (°V) for Shape, Size and Speed perception subtasks.

### Questionnaire

Table 1 summarizes the results of the SQ.

The ASQ score, calculated as the arithmetic mean of the three questions 7–9 [23], was 5.7 ± 1.4 which means that the participants liked the HVF test battery and they were particularly happy with the support information given by the instructors.

The score to the questions 1–6 showed that the mental, physical and temporal demands were well tolerated by the participants (questions 1–3), the self-estimation of the performance level (question 4) was positive, the effort needed to complete the tasks (questions 5) was also positive, but close to the middle point, the level of frustration (question 6) was low. Question 10 also had high positive feedback from the test persons, who agreed that the HVF test battery measures skills needed to correctly perceive the visual world.

## Discussion

The aims of this study were, first, to compare the image quality of two different projection approaches and, second, to conduct a pilot study to investigate the usability of the novel computer-based test to measure HVF in a hemispherical environment.

### Quality of distortion

The analysis of the two projection approaches showed that the fully measured method returns better images, in terms of quality of distortion, compared with the mathematical one.

The mathematical model presents the main advantage of being an exact solution for the distortion problem. The distortion is described by a system of two equations of fourth degree, where the intrinsic parameters can be calculated given the geometry of the system, for instance parameters like the radius and the center of the mirror, the radius and the center of the cupola and the position of the projector [24]. Among the four solutions of the equations, one and only one is acceptable for the physical problem. Once the distortion is computed, it can be easily applied to the projected images with a computational time of about 1 minute. However the mathematical model has several disadvantages, it is limited to only simple geometries and if the shapes of the cupola and the mirror are described by a higher degree polynomial, the required computational power becomes heavy and the solution would probably be approximated. Furthermore, if the mirror has a shape that cannot be described by a mathematical model or the projector is placed in an unknown position, it is impossible to compute the correct distortion. The model is also very sensitive to its own parameters, if one of those is not correctly set, the distorted images do not fit the hemispherical screen and a manual stretch is needed. In addition, the projected images have to be aligned in order to match the right position onto the cupola. But the strongest simplification of the mathematical model is that the projector is considered a point source of light, even if it is a physical device with a source screen of about 1 cm^2^. This introduces some irregularities which are more pronounced in the corner of the cupola. In order to correct them, a compensation can be applied. Under the assumption that the irregularities are small enough to be corrected with a linear compensation, a picture of the scene can be taken by a camera placed in front of the cupola and a direct comparison between this picture and the original image can be done. According to this, a map of the projection distortion can be created and used for the correction [25].

In contrast, in the fully measured approach, no mathematical hypothesis is made and the only assumption is that the observing camera is placed in the exact location where the viewer is. This approach presents the advantage of being independent from the location of the mirror and the projector, they can be ideally placed wherever it is more convenient without affecting the goodness of the distortion. Moreover the images result already aligned with the mirror and the hemisphere because they are computed directly on them. The weak point of this approach is that the positioning of the camera in front of the cupola is crucial. The camera, in fact, represents the view point of the observer and, if it is not perfectly directed towards the center of the hemisphere, the computed distortion is wrong. In this study, an infinite point of view for the observer was chosen and, as a consequence, the projected images looked like they were projected on a flat screen and not spread on a hemisphere. This was a good choice because, in the cupola, the test person has no reference point for 3D space and he performs the tasks in the same way he would on a flat screen.

Another weak point of the fully measured approach is that it requires a long calibration because the match between the two reference systems is done point by point and, in order to achieve the maximal resolution, the number of points must be equal to the resolution of the projector itself.

Processing time can be reduced by decreasing the number of projected points and interpolating the missing information, but for fine details it is not recommended. However, once the calibration is done, the images can be distorted in about two minutes.

Despite the high fidelity of the distortion shown for both methods in Table 2, the mathematical model resulted to be slightly worse than the fully measured approach because of the shortcomings mentioned above. The SSIM could be further improved for both methods considering that in the original images the shadows of the projector and the mirror are not present, and this could result in a mismatch that turns the SSIM down.

### Performance at the HVF test battery

The pilot study on the HVF test battery measured the level of accuracy of healthy subjects in comparing objects presented in the two visual hemifields, as well as the usability of the battery itself. Such a value of accuracy has never been measured before.

The single subtests for line orientation, size and shapes of objects, motion direction and speed were selected according to the differentiation of the visual analysis in the various brain areas: orientation of objects is analyzed in the Brodmann Area 17, size and shapes of objects in the “what” stream, in the Brodmann Areas 19 (anteromedial), 37 and 20; motion is analyzed in the “where” stream, in the lateral part of the Brodmann Area 19 [26]. It is clear that, according to the location of an eventual lesion, different properties of objects could result as impaired. However, in healthy subjects the HVF are intact and the only limiting factor could be the resolution of the eye, about 1 arcminute [17]. In relation to this datum, the accuracy of the test persons was slightly lower. This can be explained by taking into account that the maximum resolution of the human eye is in the fovea and it drastically decreases out of it [27], but in the HVF test battery the fovea is pointing at the marker point and not at the images under comparison. Since the images are presented at an eccentricity of 20°, our results are in line with Hunziker, who showed a decrease of the perceived sharpness of an image of 90% in correspondence of such eccentricities [28]. More in depth, the results from the Shape and Size subtasks were very similar because they investigate two aspects of the same skill, recognition of objects. A study on the redundancy of these subtasks could clarify the necessity of two separate tests but, given the purpose of this study, the integration in a clinical setting, redundancy can be helpful to generate more consistent outcomes. Similar considerations could be done for the Speed and Motion direction subtasks but, due to the different types of measure, a direct comparison is not possible. It is worth noting that the results of the Orientation and Motion direction subtasks were very different from each other although they both measure orientation. This can be explained because, according to the brain anatomy, judgment of line orientation is an easier task accomplished in the very first stages of visual perception, while motion analysis requires more specialized neurons in the posterior parietal cortex. This could be the reason why performance of the Motion direction subtask was worse than the one at the Orientation subtask.

The responses to the SQ (Table 1) showed that the HVF test battery was very well accepted by the test persons, the over-all feedback was very positive, and there were no drop-outs. In particular, question 10 measured the face validity of the subjects with regards to the battery. This is an important requirement for the usability of the test. The questionnaire measured the individual impressions of the participants, such as the mental and physical demand or the self-estimation of the performance, which are subjective but, since the test is designed to be integrated into a clinical setting, the information about the motivation of the test persons is relevant because it directly correlates with their attention to performing the tasks and, therefore, with the performance itself [29].

It is expected that patients with unilateral brain lesion in the region of the visual pathway would have worse performance compared with the age-matched healthy controls and that the performance itself negatively correlates with the degree of the lesion, the worse the performance, the higher the impairment. This can be a limitation in case of bilateral impairment, where both hemifields are affected and a comparison between affected vs. non-affected performance cannot be done. In this case, age-matched normative data could be used to judge the subject’s performance and screen for impairment. Luckily, the majority of clinical cases we see involves unilateral brain lesions (i.e. after ischemic stroke).

## Conclusions

This paper presented a usability study of a new method to measure HVF (HVF test battery). The purpose of the study was to investigate healthy volunteers’ acceptance of the developed battery rather than their performance at the visual tasks. The HVF test battery consists of five subtasks where shape, size, orientation, speed and direction of motion of objects presented in the right visual hemifield and in the left one are dynamically compared. The HVF test battery was implemented in a hemispherical environment, whose characteristic is a ±90° field of view in both horizontal and vertical directions. A hemispherical screen allows for testing of HVF in relation to peripheral and central vision, as well as in a region of the visual field still intact after eventual brain lesions. The results of the pilot study on healthy subjects are in line with literature, the usability study showed a high acceptance of the tasks by the participants. The novel HVF test battery is now ready to be tested in patients.

## Competing interests

This work was financed in parts by a research grant from the Haag-Streit Foundation.

## Authors’ contributions

GAZ carried out the technical implementation of the distortion problem, the pilot study and the analysis of the results. RM helped in the conception of the test battery. UPM carried out the clinical evaluation of the experimental procedure. TN provided important feedbacks regarding the data analyses. TNef helped in the conceptual framework of the distortion algorithm design and coordinated of the work. All authors contributed in designing the entire study, reading, correcting and approving the final manuscript.
